# Recycling of Faecal Sludge: Nitrogen, Carbon and Organic Matter Transformation during Co-Composting of Faecal Sludge with Different Bulking Agents

**DOI:** 10.3390/ijerph191710592

**Published:** 2022-08-25

**Authors:** Musa Manga, Barbara E. Evans, Tula M. Ngasala, Miller A. Camargo-Valero

**Affiliations:** 1 The Water Institute at UNC, Department of Environmental Sciences and Engineering, Gillings School of Global Public Health, University of North Carolina at Chapel Hill, 357 Rosenau Hall, 135 Dauer Drive, Chapel Hill, NC 27599, USA; 2 BioResource Systems Research Group, School of Civil Engineering, University of Leeds, Leeds LS2 9JT, UK; 3 Department of Construction Economics and Management, College of Engineering, Design, Art and Technology (CEDAT), Makerere University, Kampala P.O. Box 7062, Uganda; 4 Department of Civil and Environmental Engineering, Michigan State University, East Lansing, MI 48823, USA; 5 Departamento de Ingeniería Química, Universidad Nacional de Colombia, Campus La Nubia, Manizales 170003, Colombia

**Keywords:** carbon losses, nitrogen losses, compost maturity indices, nutrients recovery, heavy metals, micro, and macro-nutrients, faecal sludge composting

## Abstract

This study investigated the effect of locally available bulking agents on the faecal sludge (FS) composting process and quality of the final FS compost. Dewatered FS was mixed with sawdust, coffee husk and brewery waste, and composted on a pilot scale. The evolution of physical and chemical characteristics of the composting materials was monitored weekly. Results indicate that bulking agents have a statistically significant effect (*p* < 0.0001) on the evolution of composting temperatures, pH, electrical conductivity, nitrogen forms, organic matter mineralisation, total organic carbon, maturity indices, quality of the final compost and composting periods during FS composting. Our results suggest reliable maturity indices for mature and stable FS compost. From the resource recovery perspective, this study suggests sawdust as a suitable bulking agent for co-composting with FS—as it significantly reduced the organic matter losses and nitrogen losses (to 2.2%), and improved the plant growth index, thus improving the agronomic values of the final compost as a soil conditioner. FS co-composting can be considered a sustainable and decentralised treatment option for FS and other organic wastes in the rural and peri-urban communities, especially, where there is a strong practice of reusing organic waste in agriculture.

## 1. Introduction

Globally, faecal sludge management is a growing challenge, especially in the low- and middle-income countries, where delivery of sanitation services in terms of sustainable faecal sludge (FS) treatment facilities is largely still lacking [1]. Consequently, FS is collected from the on-site sanitation systems and re-used in agriculture or indiscriminately disposed of into the environment without any treatment, leading to severe public health and environmental risks [2,3].

Composting is one of the most acceptable and economically viable ways of treating FS both in rural and peri-urban communities, with the potential for critical nutrient recovery and reuse as an added advantage. However, its current operation is associated with large nitrogen (N) and carbon (C) losses, which reduces the agronomic value of the final compost [4,5,6]. Selecting the right bulking agent for co-composting with FS is essential for a successful composting process and reduction of nutrient losses such as nitrogen through ammonia volatilization [7]. In the same vein, bulking agents deserve special consideration during FS composting, because of their essential role in (i) adjusting the carbon to nitrogen (C/N) ratio, (ii) absorbing the excess moisture content and odour, and (iii) improving the physical characteristics and support structure of the composting material to provide favourable conditions for successful aerobic composting activities [8,9,10]. However, there are few scientific FS composting studies that have investigated the effect of different bulking agents on nutrient losses and compost quality.

The composting rate and quality of the final compost can be influenced by the characteristics of the sludge and bulking agent type used [10]. Several studies have been conducted on co-composting of sludge and/or organic waste with bulking agents to examine the effect of bulking agent type on the composting process and compost quality, however, contradictory results have been published [7,10,11,12,13,14,15,16]. For example, Yuan, et al. [11] reported that composting sewage sludge with cornstalk reduces the production of leachate and improves the compost quality [11]. Chang and Chen [12] showed that the composting rate and moisture content absorption capacity increased as the acidification and composting periods shorten, because of increasing the quantities of sawdust during composting of food waste. During the composting of sewage sludge, Paredes, et al. [14] observed a significant effect of bulking agent type on mineralisation and stabilisation of organic matter. Yañez, et al. [15] showed that the bulking agent types had a significant influence on the key composting parameters as well as the final compost quality, during the composting of sewage sludge. However, Doublet, et al. [13] found the bulking agent types to have very little effect on the rate of organic matter stabilisation and nitrogen in the final compost. Similarly, Tang and Katayama [16] found the bulking agents to have no effect at all on the microbial succession and composting process, especially during the early stage of co-composting cattle manure with several bulking agents (vermiculite, rice straw, waste paper, and sawdust). Therefore, further research studies are needed to clarify and update the existing literature on FS composting with bulking agents. 

Further, although studies have investigated the feasibility of FS co-composting with different bulking agents [5,6,7,8,17,18,19,20,21,22,23,24]; few attempts have been made to systematically assess FS composting process of three bulking agents in a single research to understand the effect of the different bulking agents on the evolution of organic matter, carbon, and nitrogen forms, key maturity indices, and quality of the final compost. Moreover, carbon, organic matter, and nitrogen transformation during co-composting of FS with different bulking agents using an open-air composting system is not yet well understood. Our study, therefore, investigated the effect of locally available bulking agents (i.e., sawdust, coffee husks and brewery waste) on the quality of the final compost (i.e., macro and micronutrients, and toxic elements/heavy metals), and evolution of composting temperatures, pH, EC, organic matter, carbon, Nitrogen forms, and maturity indices (i.e., carbon dioxide (CO_2_-C), Plant Growth Index, C/N ratio, NH_4_^+^-N/NO_3_^−^-N ratio) during FS composting using open-air composting system. This study findings will provide insights for decentralised treatment and sustainable management of FS and other organic wastes such as sawdust, coffee husks and brewery waste in low-and-middle-income countries, especially in the rural and peri-urban areas where there is a strong practice of reusing organic waste in agriculture.

## 2. Materials and Methods

### 2.1. Composting Materials

This study was conducted at the National Water and Sewerage Corporation (NWSC) FS treatment facility at Lubigi, Kampala, Uganda (geographical coordinates 0°18′58.18″ N latitude, 32°34′55″ E longitude, and elevation of 1223 m above sea level). The FS used in this study was collected from on-site sanitation systems (i.e., pit latrines and septic tanks) in Kampala informal settlements, and this was mixed (in a ratio of 1:2 *v*/*v*; pit latrine sludge: septic tank sludge) and dewatered on sand drying beds to about 20–35% total solids content, before being composted [25]. Sawdust, brewery waste, and coffee husks were the bulking agents tested in this study. Details for why these bulking agents were selected and used in this study are presented in our previous work [9]. Sawdust was collected from Bwaise sawmill while brewery waste and coffee husks were taken from Parambot breweries and Kyagalanyi coffee processing plant, respectively. All the collected bulking agents were sorted to remove any non-biodegradable items, prior to construction of the composting piles. Table 1 presents the key physical and chemical characteristics of the raw materials used in our study.

### 2.2. Composting Process and Sampling

Dewatered FS of about 20–35% total solids was thoroughly mixed with different sorted bulking agents. Three types of compost static piles with a volume of 3 m^3^ each, were constructed in duplicates: (i) SSD (1:2 *v*/*v*; dewatered sludge: Sawdust); (ii) SCH (1:2 *v*/*v*; dewatered sludge: Coffee husks); (iii) SBW (1:2 *v*/*v*; dewatered sludge: Brewery waste). The composting piles were aerobically composted and subjected to manual turning using a 3–7 days turning frequency. The piles were monitored for a period of 21 weeks, between April and September (relative humidity: 69–80%; air temperature: 19–26 °C), and these were protected from the rain using a composting shade roofed with clear polycarbonate roofing sheets. The moisture of all the composting piles was monitored regularly and piles were wetted as required to maintain the recommended moisture content limits of 50–65% for effective aerobic composting conditions throughout the entire composting process. The temperature within the composting piles was monitored daily at the: top (ca. 750 mm from the pile base), middle (400 mm from pile base), and bottom (200 mm from pile base), using a TFA (D-Wertheim, Model 19.2008, Sandy, UK) stainless steel body compost thermometer. 

### 2.3. Sample Collection 

A representative sample of approximately 1 kg was collected from each composting pile (See Manga, et al. [26] for a detailed procedure for collecting a representative sample from composting piles). The collected sample was then split into two equal portions where one was air-dried, ground, sieved through a <2 mm mesh sieve and used in analyses for total Kjedhal nitrogen (TKN), total organic carbon (TOC), micro and macro elements (total potassium (TK), total phosphorus (TP), magnesium (Mg), calcium (Ca), sodium (Na), iron (Fe), manganese (Mn)) and toxic heavy metals (copper (Cu), nickel (Ni), lead (Pb), cadmium (Cd), chromium (Cr), and zinc (Zn)). While the other portion of the fresh sample was preserved at 4 °C and analysed within <4 h for pH, EC, moisture content, ammonium-nitrogen (NH_4_^+^-N), nitrates (NO_3_^−^-N), plant growth index (PGI) and CO_2_-C respiration rate [27,28]. Compost samples were collected on day 0 and weekly, until the end of the composting period, and these were taken to Bugolobi NWSC central laboratory and Makerere University, School of Agriculture’s Soil Science Laboratory, Kampala, Uganda for analysis.

### 2.4. Analytical Methods

Moisture content was computed from the difference in the sample initial and final (after oven drying at 105 °C for 24 h) weights, following the procedure stipulated in Okalebo, et al. [27]. The oven dried samples (at 105 °C for 24 h) were then ashed in a muffle furnace for 8 h at 550 °C to quantify the total organic matter content, which was the difference between sample weight before and after combustion [27]. The pH and EC measurements were conducted in water-soluble extract of 1:10 (*w*/*v*) ratio, using a pH electrode and EC probe of HACH multi-meter (sensION MM374), respectively. Compost samples were analysed for organic carbon by oxidation using potassium dichromate [27,29]. Nitrogen content was analysed following the kjeldahl method for TKN [27,30], while NO_3_^−^-N and NH_4_^+^-N were extracted with 0.5 K_2_SO_4_ in 1:10 (*w*/*v*) from fresh compost samples and determined by spectrophotometric methods according to procedures reported in the literature [27,31]. organic matter (OM), nitrogen, and TOC losses were computed according to Equations (1)–(3) [32]:

OM loss (%) = 100 − 100[X_1_(100 − X_2_)]/[X_2_(100 − X_1_)] (1)

N_T_ loss (%) = 100 − 100[(X_1_N_f_)/(X_2_/N_i_)] (2)

TOC loss (%) = 100 − 100[(X_1_TOC_f_)/(X_2_/TOC_i_)] (3)

where X_1_ and X_2_ are the initial and final ashes content (%); N_i_ and N_f_ are the initial and final content of total nitrogen (TKN + NO_3_^−^-N). TOC_i_ and TOC_f_ are the initial and final content of TOC.

The microbial respiratory activity in compost samples was measured based on CO_2_-C evolution rate conducted in closed bottles according to Öhlinger [33] soil respiration techniques, but with some modifications made to techniques based on similar soil respiration procedures reported in the literature [34,35]. CO_2_-C was trapped in an alkaline solution (KOH), which was then titrated with a HCl solution (0.5*M*). CO_2_-C production rate was assessed and expressed as mg CO_2_-C per mass of organic matter (as Volatile Solids–VS) per day [35]. Air-dried samples for toxic elements/heavy metals (Cu, Ni, Pd, Cd, Cr, and Zn) analysis were digested using mixed acid digestion (HClO_4_ + HNO_3_ + H_2_SO_4_), and the wet-digest was used in determining the concentrations of each element using an atomic absorption spectrophotometer [27,36]. Samples for macro and micronutrients (K, TP, Mg, Ca, Na, Fe, Mn) analysis were digested using sulphuric acid-hydrogen peroxide [27,36] and the wet-digest was used in determining concentrations of Mg, Ca, Mn, and Fe content using atomic absorption spectrophotometer (Agilent 240Z AA (200 series AA) with PSD 120 and GTA 120, model, AA-01-0400, Agilent Technologies, Mississauga, ON, Canada), and K and Na concentrations using flame photometer (model 420 flame photometer, Sherwood Scientific, Cambridge, UK). However, total phosphorus was determined on the wet digests using ascorbic acid method [27]. Table S1 in the supplementary information presents a summary of the analytical methods and laboratory instruments used in the analysis. 

The plant growth index (PGI) experiment was performed in a greenhouse of the department of soil science at Makerere University, Kampala, Uganda. The PGI test was conducted using a mixture of compost and peat in ratios of 0%, 25%, 50%, 75% and 100% (*v*/*v*). The mixture was placed in 1 litre plastic pots. About 10 tomato seeds (Nuru F1 hybrid *Lycopersicon esculentum*) were evenly planted in each pot at a depth of about 10 mm in the mixtures and then well moistened. All experiments were conducted in triplicates. The pots were covered with a transparent plastic film, which was removed after 3–4 days when 50% of the sown seeds in the growing medium had emerged through the surface. The pots treated with 0% compost (100% peat) were used as the control. In the greenhouse, the ambient temperature ranged between 18–26 °C, with natural daylight of 12 h during the experiment period. Every 1–2 days, the plants were checked and watered with an equal volume of distilled water. The plant growth experiment was conducted for 21 days. At the end of the experiment, the germinated seedlings were harvested by cutting the shoot close to the substrate surface. The harvested shoots were then oven-dried to a constant weight at 70 °C for 72 h and weighed (accuracy ± 0.01 g). The PGI value was then expressed as the ratio of the mean of the total shoot weight of the treatments (25%, 50%, 75%, and 100% of compost) to the mean of the weight of the control samples (0% of compost) using Equation (4) [37,38].

(4) $$\mathrm{PGI}\left(\%\right)=\frac{\mathrm{Mean}\;\mathrm{total}\;\mathrm{dried}\;\mathrm{shoot}\;\mathrm{weight}\;\mathrm{of}\;\mathrm{the}\;\mathrm{germinated}\;\mathrm{seeds}\;\mathrm{in}\;\mathrm{the}\;\mathrm{treated}}{\mathrm{Mean}\;\mathrm{total}\;\mathrm{dried}\;\mathrm{shoot}\;\mathrm{weight}\;\mathrm{of}\;\mathrm{the}\;\mathrm{germinated}\;\mathrm{seeds}\;\mathrm{in}\;\mathrm{the}\;\mathrm{Control}}\times 100$$

### 2.5. Statistical Analysis

The results were reported as average values ± standard deviation of duplicates and subjected to statistical analysis using IBM SPSS version 21.0 software (IBM, New York, NY, USA). Data were analysed using the non-parametric Friedman test for testing the significance of differences amongst the mean values, with a 95% confidence level. Spearman’s rho test was used for evaluating the relationships between variables. *p* < 0.05 was set as the statistical significance.

## 3. Results and Discussion

### 3.1. Temperature Evolution

**Figure 1:**
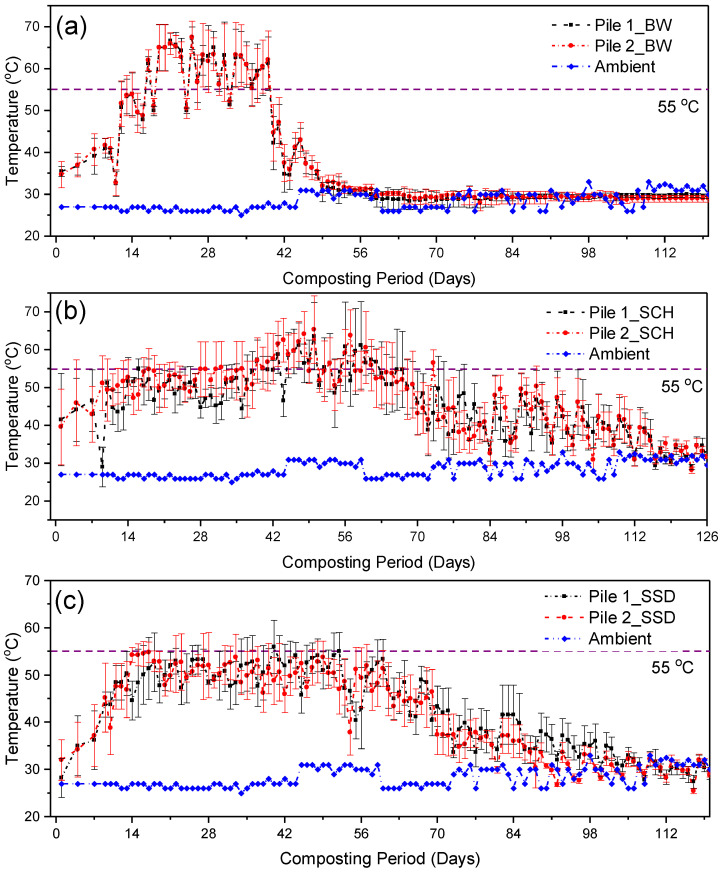
Temperature evolution during composting of faecal sludge-FS with: (**a**) brewery waste-SBW; (**b**) coffee husks-SCH; and (**c**) sawdust-SSD. In (**a**–**c**) error bars represent the standard deviation of the bottom, centre, left side, and right-side pile temperatures.

The temperature evolution of the three bulking agent composting piles is shown in Figure 1. As is common with all composting processes, we observed three different temperature phases in our study. Firstly, an initial mesophilic phase (27–44 °C), which lasted in the SCH, SBW and SSD composting piles for about five, 12, and 10 days, respectively, followed by a thermophilic phase (45–72 °C), which lasted for about 97 days (SCH), 29 days (SBW) and 59 days (SSD) in the composting piles. The thermophilic phase is referred to as the active stage, characterised by intense microbial activities, presence of readily degradable carbon, high temperatures, and rapid degradation rates of organic matter [39]. We further observed a second mesophilic and/or curing phase which immediately followed the thermophilic phase once the supply of readily available carbon became limited. This phase was characterised by mesophilic conditions with low temperatures (<45 °C) and low rates of mineralisation and humification of organic matter. The SSD and SBW piles exhibited longer initial mesophilic phase than SCH piles, and this might have been due to the shortage of easily available biodegradable carbon sources. 

In our study, we observed that SSD and SCH piles recorded longer thermophilic phase duration than SBW piles (See Figure 1a–c). This was probably due to their water retention capacity, high carbon content, and availability of higher specific surface for microbial attack, which all support microbial activities for a prolonged period. Such a finding was also observed by Leconte, et al. [40] during the co-composting of sawdust with poultry manure. Irrespective of the longer thermophilic phase in SSD and SCH piles, the SBW piles exhibited the highest composting temperatures in all sections of the composting piles (67–70.2 °C) during the thermophilic phase (See Figure 1b); however, these thermophilic conditions were short-lived. This response can be explained by the intensive microbial activities during early composting stages, which might have contributed to the faster consumption and depletion of the easily biodegradable carbon in the SBW piles than in other composting piles, thus a drop in the microbial activities and temperatures during the later stages. Previous researchers have similarly found microbial activities to be regulated by the presence of easily biodegradable carbon sources during composting [28,41]. 

In our study, the composting process was considered complete once the composting temperatures reached the ambient temperature, and SBW piles exhibited the shortest composting period of 57 days compared to SSD (with 109 days) and SCH (with 136 days) (Figure 1a–c). A detailed discussion on the temperature evolution of these composting piles is presented in a separate note elsewhere [9]. We observed evidence that bulking agent types have a statistically significant effect (*p* = 0.0001) on the evolution of composting temperatures of FS. All the composting piles attained and sustained the optimum conditions recommended for effective and complete sanitization of the composting material. 

### 3.2. pH Evolution

**Figure 2:**
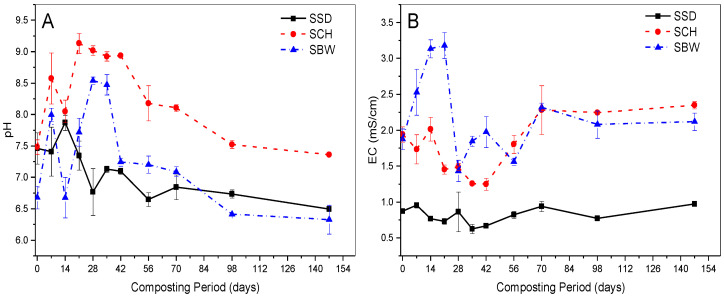
Evolution of: (**A**) pH; and (**B**) electrical conductive (EC) during the composting of Faecal Sludge-FS with Brewery waste-SBW, Coffee husks-SCH, and Sawdust-SSD with different bulking agents. Error bars represent the standard error of *n* = 2.

SSD, SCH, and SBW composting piles exhibited an initial increase in pH values, reaching peak values of 7.9, 9.1, and 8.5 at 14, 21, and 28 days; followed thereafter by a gradual decrease as the composting process progressed but with slight fluctuations, reaching final values of 7.4, 6.3 and 6.5, respectively, by the end of the composting process (See Figure 2A). The final pH values attained in this study are comparable to those reported in the literature [40,42]. The increase in the pH values observed especially during the thermophilic phase may be explained by ammonia production, which is linked to the decomposition of proteins within the composting material, mineralisation, and ammonification of organic N, as well as NH_3_-N solubilisation. This hypothesis was confirmed by a significant positive correlation between the evolution of pH and NH_4_^+^-N during the composting of SSD (*p* = 0.006, R^2^ = 0.32), SCH (*p* = 0.001, R^2^ = 0.42), SBW (*p* = 0.012, R^2^ = 0.28) piles. A similar observation was reported by Sánchez-Monedero, et al. [43] and Eklind and Kirchmann [44] during the composting of similar feedstock. 

We observed that all composting piles exhibited decreases in pH values during the thermophilic phase (See Figure 2A). This behaviour may be explained by ammoniacal nitrogen volatilisations and precipitation of carbonate in the form of calcium carbonate [44]. In this study, SSD piles exhibited generally lower pH values than SCH and SBW piles throughout the composting process. This may be because they contained a high proportion of sawdust which is generally acidic in nature. Similar observations have been reported by Huang, et al. [45] during the composting of pig manure with sawdust. Our study results indicate that the three bulking agents tested have a significant effect (*p* = 0.0001) on the pH evolution during FS composting. 

### 3.3. Electrical Conductivity (EC)

In composting, EC values reflect the salinity of compost, and some authors have suggested it as an index for evaluating the composting process efficiency as well as the compost maturity or stability [28]. In our study, the initial average EC values of the SSD (0.87 mS/cm), SCH (1.94 mS/cm), and SBW (1.88 mS/cm) composting piles gradually increased but with fluctuations, especially in the thermophilic phase to final EC values of 0.98, 2.35 and 2.12 mS/cm, respectively, at the end of the composting process. These increases in EC values may be attributed to the mineralisation of organic matter and the concentration effect of soluble salt resulting mainly from the net weight loss. This behaviour was also observed by Cáceres, et al. [41] and Fang and Wong [46] during the composting of the solid fraction of cattle slurry and sewage sludge with lime amendment, respectively. 

In Figure 2B, SBW composting piles exhibited the highest average EC values (1.44–3.18 mS/cm) especially during the thermophilic phase, followed by SCH piles (1.25–2.35 mS/cm) and SSD piles (0.63–0.98 mS/cm) with the lowest. This response can be attributed to the active decomposition phase, which may have contributed to the significant release of ions bound to the organic matter into water-soluble form during these composting periods [38]. In our study, we noted that SSD piles exhibited the lowest EC values throughout the composting process (Figure 2B). This behaviour is thought to have been due to the dilution effect of EC values in the FS since sawdust is characterised by considerably low EC values.

Our results indicate that the studied bulking agent types have a statistically significant effect (*p* = 0.0001) on the EC values during FS co-composting. The final EC values (0.98–2.35 mS/cm) achieved in this study compare well with those published in the literature [46,47]. We observed that the final EC values of our FS compost were below the threshold value of 3.0 mS/cm at which no adverse effect can be exerted on the growth of slightly salt-tolerant plant species [48]. 

### 3.4. Changes in Total Organic Carbon and Organic Matter

**Figure 3:**
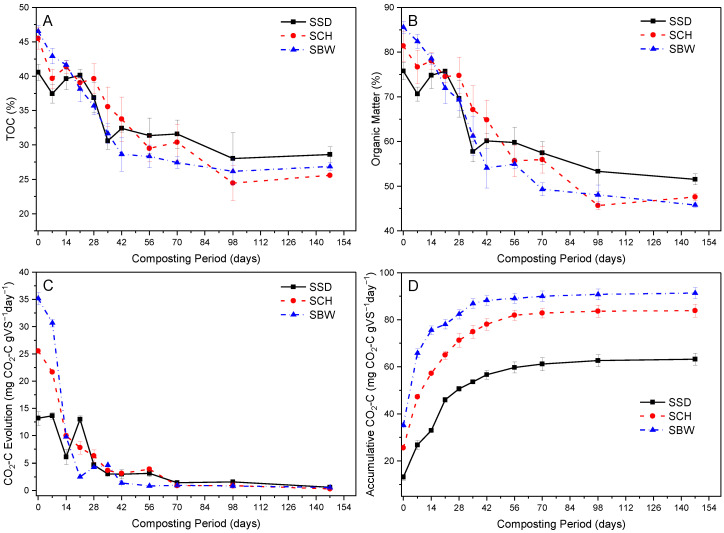
Changes in: (**A**) total organic carbon; (**B**) organic matte; (**C**) carbon dioxide rate per organic matter (CO_2_-C/TVS); and (**D**) cumulative carbon dioxide per organic matter during the composting of faecal sludge-FS with brewery waste-SBW, coffee husks-SCH, and sawdust-SSD. Error bars represent the standard error of *n* = 2.

In Figure 3B, SBW, SSD and SCH organic matter decreased from the initial average values of 85.6%, 75.8% and 81.4% to 45.8%, 51.6% and 47.6% after 21 weeks of composting, signifying OM losses of 87.8%, 65.7% and 79.2% of the initial OM, respectively, (See Supplementary Information Figure S1). Similar trends were observed in the evolution of average TOC decomposition with the SBW, SSD, and SCH composting piles recording a final TOC average value of 26.9%, 28.6%, and 25.6%, respectively, at the end of the composting period (Figure 3A). The substantial reduction in TOC and OM content observed in this study indicates that significant mineralisation of organic carbon and organic matter occurred, and thus a reduction in volume and weight of the composting piles. 

In our study, we noted that during the composting of SBW, SCH and SSD feedstock, TOC of about 197 kg, 199 kg, and 120 kg was converted to CO_2_-C, and this represents TOC loss of approximately 85.8%, 79.2%, and 65.7%, respectively. These results indicate that high proportions of organic carbon were lost in the form of carbon dioxide during the co-composting of FS with different bulking agents. This fact was confirmed by a very strong negative correlation between the evolution of TOC and CO_2_-C respiration rate during the composting of SSD (*p* = 0.0001, R^2^ = 0.755), SCH, (*p* = 0.0001, R^2^ = 0.841), SBW (*p* = 0.0001, R^2^ = 0.828) composting piles. 

SBW piles and SCH piles exhibited higher TOC and OM losses than SSD piles during composting (See Supplementary Information Figure S1). A similar observation was made by Dias, et al. [49] during the composting of poultry manure with coffee husks or sawdust. This might have been because the SBW and SCH feedstock contained more proportions of easily biodegradable organic substances than SSD feedstock. Alternatively, the higher organic matter losses exhibited by such piles can be attributed to the chemical composition of coffee husks and brewery waste. For example, Pandey, et al. [50] reported coffee husks to contain a considerable amount of Proteins (9.2%) and carbohydrates (57.8%), of which about 18–25% by weight - of this coffee husks carbohydrates can be easily hydrolysed as glucose [51]. Therefore, high organic matter losses exhibited by piles containing such wastes may be attributed to the high temperatures and intense microbial activities which may have facilitated the hydrolysis of some complex polysaccharides present in such wastes [49].

In the same vein, we noted that SSD piles recorded higher organic matter and TOC content than SCH and SBW throughout the entire composting period, which implies that significantly low OM decomposition occurred in such piles. This might be because the SSD piles might have contained large proportions of more recalcitrant decomposable carbon compounds such as lignin, and cellulose, which are not easily biodegraded by microbial during composting and thus resulting into lower TOC and OM decomposition rates. This observation has also been documented by several researchers [49].

Our study results indicate that the tested bulking agent types have a significant effect on the organic matter losses (*p* = 0.0001) and the composting period during FS composting. We observed that the OM and TOC content in SBW piles stabilised faster (after 8 weeks) than in SSD and SCH piles (after 14 weeks), and this correlated with the composting temperatures profiles (Figure 1a–c). This study findings suggest that co-composting of FS with brewery waste, would reduce the composting period by 6 weeks. 

### 3.5. Evolution of Carbon Dioxide (CO_2_-C)

In this study, SBW, SSD, and SCH piles exhibited a gradual decrease in the CO_2_-C respiration rate as the composting process progressed, reaching stable low average values of 0.83, 0.6 and 0.84 mg CO_2_-C gVS^−1^day^−1^ within a composting period of eight, 21, and 10 weeks, respectively (Figure 3C). These low average respiration rates reached in all the monitored piles towards the end of the composting process indicate that the compost was generally stable. The CO_2_-C concentrations attained in the current study are in agreement with those published by other researchers [52].

As seen in Figure 3C, all composting piles exhibited considerably high CO_2_-C respiration rates during the first two weeks of composting. This response can perhaps be attributed to the presence of fresh organic substances or a high quantity of easily biodegradable organic carbon at the beginning of the composting process, which facilitated significant growth and activities of microorganisms within piles. Although, some previous studies [15,47] have reported a significant association between the CO_2_-C respiration rate and the composting temperature, we never observed this in our study. This can perhaps be attributed to the heat accumulation phenomenon within the composting solid material, which eventually results in a further rise in the composting temperatures, yet the microbial activities measured by CO_2_-C respiration rate are already decreasing [53]. 

As seen in Figure 3D, SBW piles exhibited higher cumulative CO_2_-C respiration rates than SCH and SSD piles. This implies that SBW piles contained considerably higher quantities of easily biodegradable organic carbon and favorable conditions, which supported the greater growth and activities of micro-organisms in such piles. This also correlated well with higher composting temperatures recorded in these piles. In our study, we observed increases in the CO_2_-C respiration rate as the composting process progressed, especially in the SSD composting piles. This response can be attributed to the degradation of hardly biodegradable organic carbon sources. A similar phenomenon was observed by other researchers [54]. 

Previous authors have identified CO_2_-C evolution as a sensitive and reliable indicator for assessing the efficiency of the composting process as well as the compost maturity since it is directly connected to aerobic biological activities [53]. Studies have documented <2 mg CO_2_-C gVS^−1^day^−1^ and <1 mg CO_2_-C gVS^−1^day^−1^ as CO_2_-C threshold values for very mature compost [55,56]. According to such criterion, SBW piles reached maturity after a composting period of 8 weeks, followed by SCH and SSD piles with 10 and 21 weeks, respectively. This result suggests that bulking agent types influence the composting periods of FS. In the same vein, our results indicate that the studied bulking agent types have a statistically significant effect (*p* = 0.0001) on the CO_2_-C evolution during the composting of FS.

### 3.6. Changes in Nitrogen Forms

#### 3.6.1. Total Nitrogen (TN) and Organic Nitrogen 

**Figure 4:**
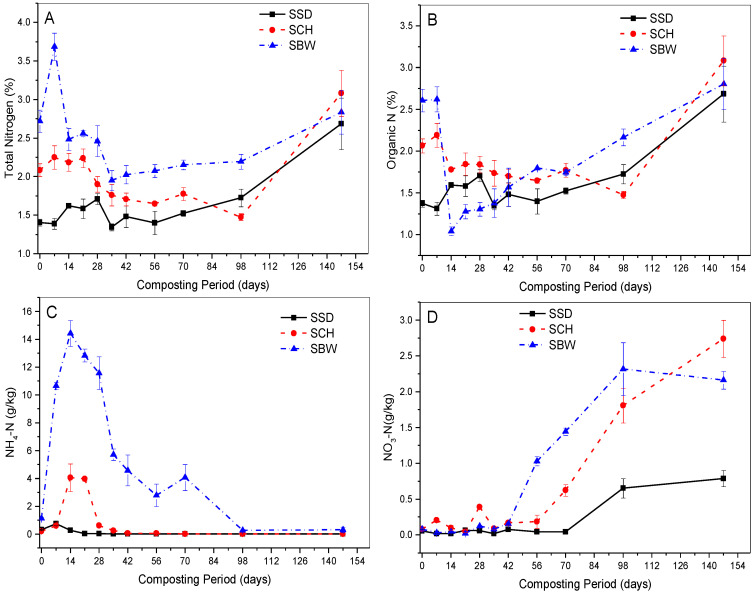
Evolution of: (**A**) total Nitrogen (TN); (**B**) organic nitrogen; (**C**) ammonium-nitrogen (NH_4_^+^-N); and (**D**) nitrate-nitrogen (NO_3_^−^-N) during the composting of faecal sludge-FS with brewery waste-SBW, coffee husks-SCH, and sawdust-SSD. Error bars represent the standard error of *n* = 2.

The effect of the three bulking agent types was observed in the TN and organic N dynamics throughout the composting process. In Figure 4A, the TN content of SBW, SSD and SCH piles gradually increased but with significant fluctuations from original values of 3.1%, 1.4%, and 2.9% to final values of 2.8%, 2.7%, 3.1%, respectively, at the end of 21 weeks composting period; representing a 90.7% (SSD), 47.3% (SCH) and 4.4% (SBW) increase in the TN concentrations on the initial dry weight. Similar trends were observed in the evolution of organic nitrogen with the SBW, SSD, and SCH piles recording final average values of 2.8%, 2.7%, and 3.1%, respectively, at the end of the composting process (Figure 4B). This increase in the TN and organic N concentrations on an initial dry weight basis might be explained by the concentration effect resulting from the significant decomposition of labile organic carbon compound, which may have led to a reduction in the dry weight and volume of the composting material. This behaviour has also been documented in several composting studies [54,57]. 

As shown in Figure 4A,B, all composting piles exhibited some decreases in TN and organic N concentrations during composting. This might be attributed to nitrogen mineralisation and transformation to NH_4_^+^-N, especially during the thermophilic phase, and later to NO_3_^−^-N during the maturation phase. We observed evidence that the lowest organic nitrogen concentrations exhibited by piles (especially in SBW piles) synchronised well with the highest NH_4_^+^-N concentration recorded (See Figure 4B,C), which confirms that some fractions of organic nitrogen were mineralised to produce NH_4_^+^-N. Similar, behaviour was also found by other researchers [6,57]. 

Interestingly, N losses were noted from all piles when the TN increase was computed on an ash content basis. The SBW, SCH and SSD piles recorded N-losses of about 72.5%, 48.2% and 2.2%, respectively (See Supplementary Information, Figure S1). The N-losses exhibited by all composting piles in our study are thought to have been due to the strong degradation of organic substances by intensive microbial activities. This hypothesis was confirmed by a statistically significant negative association between evolution of CO_2_-C and N-loss throughout the composting process (SSD piles (*p* = 0.04, R^2^ = 0.19); SCH piles (*p* = 0.0001, R^2^ = 0.46); SBW piles (*p* = 0.0001, R^2^ = 0.68)). Our results suggest that to a certain extent the microbial activities are responsible for the N-losses during composting. Benito, et al. [58] also observed a similar behaviour during the composting of pruning waste. On the other hand, the higher temperatures (>40 °C) and pH (above 7) especially during the thermophilic phase might also have partly enhanced this N-losses process through NH_3_-N volatilisation [59].

**Figure 5:**
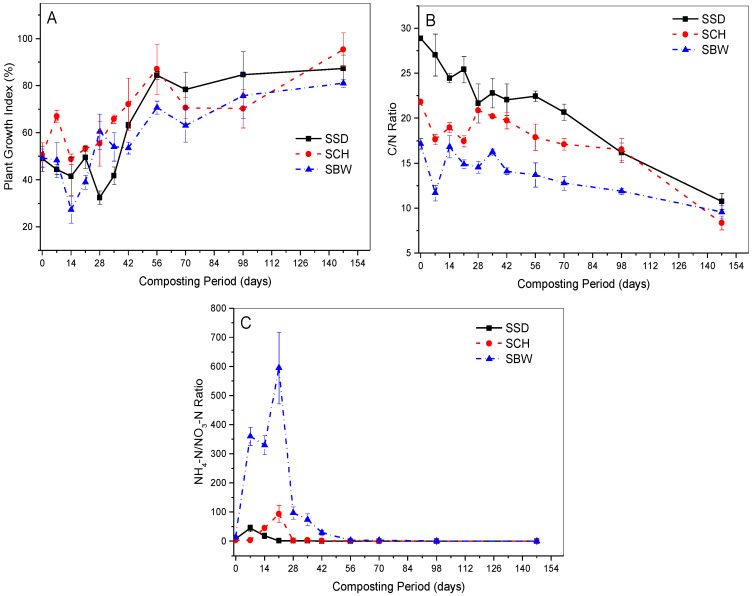
Changes in: (**A**) plant growth index; (**B**) carbon/nitrogen (C/N); and (**C**) ammonium-nitrogen/nitrate (NH4^+^-N/NO_3_^−^-N) during composting of faecal sludge-FS with brewery waste-SBW, coffee husks-SCH, and sawdust-SSD. Error bars represent the standard error of *n* = 2.

We observed that SBW piles exhibited the highest initial average TN values (3.1%) and N-losses (72.5%) followed by SCH and SSD piles. The higher nitrogen content and very low C/N ratio (<20:1) observed in the SBW feedstock (Figure 5B) at the beginning of the composting process is thought to have been responsible for the huge N-losses exhibited by these piles, mainly in the form of NH_3_-N volatilisation. Similarly, several studies have found the degree of N-losses during composting to mainly depend on the characteristics of the starting feedstock, especially C/N ratio [60,61]. 

It is noteworthy that the SSD piles exhibited the lowest initial average TN value of 1.4% and N-losses of only 2.2% at the end of the composting process. This can be attributed to the enhancement of N immobilisation in microbial biomass due to the use of the sawdust (in the starting feedstock) that contains a high content of lignocellulosic material. This phenomenon was also observed by Leconte, et al. [40] who found piles containing sawdust to have conserved more nitrogen than rice hull piles. 

This study results indicate that the bulking agent types have a statistically significant effect (*p* = 0.0001) on the organic nitrogen mineralisation and TN evolution during FS composting. Importantly, our results suggest that composting of FS with sawdust or coffee husks reduces N-losses by 24–70%. 

#### 3.6.2. Ammonium-Nitrogen (NH_4_^+^-N) 

In Figure 4C, the NH_4_^+^-N trends of the three types of bulking agent compost differed significantly (*p* = 0.0001). The average NH_4_^+^-N concentrations of SCH and SSD composting piles were considerably low during the early composting periods, and these increased reaching peak values after 2 and 1 weeks of composting; followed by a decrease thereafter to a mean low value of 0.010 and 0.009 g/kg, respectively, at the end of the composting process. Similarly, the NH_4_^+^-N concentrations of the SBW piles increased rapidly from the initial mean value to peak values within 2 weeks of composting, and this then decreased to a mean low value of 0.310 g/kg after a composting period of 21 weeks. Our NH_4_^+^-N final concentrations and evolution trends align well with those published in the literature [62]. 

In Figure 4C, it can be noted that SCH piles did not exhibit an initial increase in the NH_4_^+^-N concentration until the 7th day. This can be explained by the delay in attaining thermophilic temperatures in such piles, which clearly indicates delayed microbial and decomposition activities as well as organic matter and nitrogen mineralisation. In our study, we observed that all the composting piles exhibited an increase in NH_4_^+^-N concentrations during the early stages of composting. This response may be attributed to the formation of NH_3_-N from organic nitrogen as the increase in NH_4_^+^-N was observed to be coupled with a slight decrease in organic N (Figure 4A,C). 

As shown in Figure 4C, SBW piles exhibited considerably higher NH_4_^+^-N content than SSD and SCH piles throughout the composting process; and this might have been due to the higher initial easily decomposable nitrogenous substances with a lower C/N in the starting SBW feedstock. Surprisingly, during the composting of SSD piles, comparatively low NH_4_^+^-N concentrations of approximately 0.28 g/kg were recorded during the thermophilic phase where the pH (was >7.0) and composting temperatures were considerably still high (>40 °C), yet studies by Bishop and Godfrey [63] found high temperatures (>40 °C) and high pH (>7.0) to have been responsible for the significant nitrogen losses via NH_3_-N volatilization. This may probably have been due to the significant NH_3_-N losses from the porous composting mixture to the atmosphere as the convective air flows through the composting piles. It is likely that the SSD composting material may have lost its non-porousness physical structure and bulk density during intensive composting periods and regular turning. This phenomenon was also observed by Changa, et al. [52] during the composting of dairy manure with straw.

In all the composting piles, NH_4_^+^-N concentration decreased to low mean values after the composting temperatures had decreased to mesophilic conditions (<40 °C) (Figure 1a–c). This behaviour can be attributed to the high rates of NH_4_^+^-N losses via the nitrification process (NO_3_^−^-N) [64]. This observation was confirmed by a statistically significant and meaningful negative correlation (SSD (*p* = 0.023, R^2^ = 0.23); SCH (*p* = 0.0001, R^2^ = 0.48); and SBW (*p* = 0.0001, R^2^ = 0.64)) between the evolution of NH_4_^+^-N and NO_3_^−^-N content during composting. 

Previous studies have recommended the use of NH_4_^+^-N as an indicator for assessing the composting process efficiency as well as compost stability or maturity. In the same vein, the threshold NH_4_^+^-N value of <0.3 g/kg has been suggested for mature or stable compost [65]. In our study, this threshold value was reached and surpassed by all piles before the end of the composting process, which implies that the compost was mature. 

#### 3.6.3. Nitrate-Nitrogen (NO_3_-N)

In Figure 4C,D, we observed that as the concentrations of NH_4_^+^-N decreased during the composting of SBW, the NO_3_^—^N concentrations increased gradually reaching an average value of 2.32 g/kg on the 14th week, but this dropped to 2.16 g/kg by the end of 21 weeks composting period. However, in SCH and SSD piles, NO_3_-N concentrations gradually increased with fluctuations after 4 and 10 weeks of composting, reaching final values of 2.7 g/kg and 0.79 g/kg, respectively, at the end of the composting period. 

In this study, SBW and SCH piles exhibited higher NO_3_-N content than SSD piles at the end of the composting process (Figure 4D). This might have been due to the higher initial TN concentration that was present in the SBW and SCH feedstock. During the early composting stages, the rate of nitrification was generally low in all the composting piles (Figure 4D). This might probably be because of high ammonia concentrations, high temperatures, and pH values observed during the thermophilic phase, which might have impeded the growth and activities of nitrifying bacteria. This observation was supported by a significant negative correlation between the evolution of pH and NO_3_-N concentration during the composting of SSD piles (*p* = 0.007, R^2^ = 0.31), SCH piles (*p* = 0.048, R^2^ = 0.18) and SBW piles (*p* = 0.022, R^2^ = 0.24); and a strong negative correlation between the evolution of NH_4_^+^-N and NO_3_-N (see the NH_4_^+^-N section). Previous composting studies have also found the high temperatures (>40 °C), high ammonia concentrations and high pH to have strongly hindered the growth and activities of the nitrifying bacteria (nitrification process), especially during the thermophilic phase [66] 

As shown in Figure 3D, we observed some decreases in the NO_3_^−^–N concentrations during the early composting periods, especially in the SCH piles. This might have been due to NO_3_^−^-N losses via microbial denitrification from NO_3_^−^-N to NO, N_2_O, and N_2_; and this may have occurred in some isolated zones within the composting material, which may have turned or become anaerobic. This is in agreement with Tiquia and Tam [57] who found microbial denitrification to have been responsible for NO_3_^−^-N losses - as considerably high denitrifying bacteria populations were observed during early composting periods. 

Our study results demonstrate that the bulking agent types have a statistically significant effect (*p* = 0.0001) on NO_3_^−^-N evolution during the composting of FS. The final NO_3_^−^-N concentrations observed in this study conform very well with 1.746–3.019 g/kg and 0.9–3.03 g/kg published in Paredes, et al. [32] and Leconte, et al. [40]. Fuchs, et al. [65] recommended a NO_3_^−^-N threshold value of 0.066 g/kg TS or 0.04 g/kg FS for compost application in the garden, and the final NO_3_^−^-N values attained in this study were greater than this threshold value. This implies that compost was mature and safe for use in agriculture. 

### 3.7. Compost Maturity

#### 3.7.1. Plant Growth Index (PGI) 

In Figure 5A, SSD and SBW piles exhibited a decrease in the initial PGI value from 49% and 49.3% to the lowest values of 32.5% and 27.3% on day 28 and 14, respectively. This was then followed by a gradual increase but with slight fluctuations, reaching final values of 87.2% and 81.0%, respectively, by the end of the composting process. On the contrary, SCH piles showed an initial increase in the PGI values within the first week, followed by a decrease reaching the lowest value of 48.7%; then a gradual rise thereafter to the final value of 95.3% at the end of the composting process (Figure 5A). It was noted that the lowest PGI values recorded synchronised with the high NH_4_^+^-N concentration (Figure 4C and Figure 5A). 

Our study findings demonstrate that PGI values of the three bulking agents composting piles, generally increased as the composting process progressed. This response can be explained by the decomposition of phytotoxic compounds (such as NH_4_^+^-N, short chain volatile fatty acids) present in the composting material. In Figure 5A, significant decreases in the PGI values were observed in all composting piles, especially during the early stages of composting. This may have been due to the release of high NH_4_^+^-N concentration during the active stages of composting. This observation was supported by a strong negative relationship observed in all composting (SSD (*p* = 0.002, R^2^ = 0.40), SCH (*p* = 0.0001, R^2^ = 0.48), SBW (*p* = 0.0001, R^2^ = 0.54)) between evolution of PGI and NH_4_^+^-N concentrations. A similar phenomenon was observed by Tiquia, et al. [67] during the composting of spent pig-manure sawdust litter, who found NH_4_^+^-N to have been the major factor responsible for the phytotoxicity of plant species studied. On the other hand, the decrease in the PGI may also be linked to the degradation of organic matter, which results in the production or release of short-chain organic acids (especially acetic, valeric and propionic acids) [39,68].

During the late stages of composting, all the composting piles exhibited a significant increase in the PGI values, which implied that the phytotoxic substance had disappeared, and the compost was generally phytotoxic free and mature. The literature has documented the germination index/plant growth index as one of the most reliable and sensitive indicators for evaluating compost phototoxicity and maturity [68]. PGI threshold value of ≥80% has been suggested for phytotoxic-free and mature compost [69]. In the present study, this threshold value was reached and exceeded after a composting period of 8 weeks by SSD and SCH piles. However, the compost was still immature at this stage since further fluctuations (drops) in PGI values below the threshold value were observed as the composting process progressed, until 14 and 21 weeks, respectively, when they stabilised. Similarly, the SBW composting piles reached the threshold value after a composting period of 21 weeks and this implied that the compost was mature. It is very interesting to note that according to this PGI threshold value criterion, the SBW compost reached maturity after 21 weeks composting period, yet the composting temperature and other maturity indices had indicated that the SBW compost had stabilised or matured about 4 weeks earlier. This result suggests that there is no single index that can be used solely for accessing the efficiency of the composting process and compost maturity. However, the delay of the SBW piles in reaching the PGI threshold value can be attributed to the high NH_4_^+^-N concentrations observed in such piles, which may have inhibited plant growth. The PGI results also suggest that the bulking agent types had a significant effect (with *p* = 0.016) on the composting period of FS. 

The final PGI values of 81.0–95.3% recorded in this study are in line with those published by other authors [68]. However, these PGI values are higher than the 60% GI values (open cress) reported by Cofie, et al. [6] during the composting of a similar feedstock. This discrepancy in the PGI values results could have been due to the differences in the methodologies used as well as the type and characteristics of the soil used for the control samples. In the present study, peat soil was used for the control samples while in the latter, steamed sterilised soil (for 3 h at 100 °C) stored for one year was used.

#### 3.7.2. C/N Ratio

In this study, SSD piles recorded the highest mean initial C/N ratio that gradually declined from 28.9 to 10.8 after 21 weeks of composting. Similarly, in SCH piles, the initial C/N ratio dropped gradually from 21.84 to 7.57 within the composting period of 21 weeks. However, the SBW piles recorded a relatively low mean initial C/N ratio of 15.0, which decreased to 9.55 by the end of the composting process (Figure 5B). 

Although the three bulking agent compost types recorded a significant difference in the C/N ratio values (*p* = 0.0001), they all exhibited a similar C/N ratio evolution pattern, with a gradual decrease as the composting process progressed. The gradual decrease in the C/N ratio has been well documented in the literature and attributed to the decrease in organic carbon and increase in total nitrogen as the composting process progresses [60,68]. However, the exhibited significant difference in the C/N ratio values of the three bulking agent compost types may probably have been due to the difference in the proportions of easily biodegradable carbon sources within the composting feedstock. 

Several researchers have suggested C/N threshold values for stable and mature compost. In the same vein, an optimum C/N value of <12 was proposed by Iglesias Jiménez and Pérez García [70] for compost with a good grade of maturity. This threshold value was reached by SSD, SCH, and SBW piles after a composting period of 21, 21 and 14 weeks, respectively. This implied that the compost was mature and therefore, can be reused in agriculture. Our study results indicate that the bulking agent types have a statistically significant effect (*p* = 0.0001) on the compost maturity and composting periods of FS. The C/N ratio values attained in our study are in line with those published in the literature [71]. 

#### 3.7.3. NH_4_^+^-N/NO_3_^−^-N Ratio

Figure 5C presents the evolution of NH_4_^+^-N/NO_3_^−^-N ratio during the composting of FS with three bulking agent types. In this study, the NH_4_^+^-N/NO_3_^−^-N ratio of SSD, SCH, and SBW composting piles decreased gradually as the composting process progressed, reaching relatively low and stable values of 0.011, 0.003 and 0.15, respectively, by the end of the composting period (Figure 5C). This response can be attributed to the drop in NH_4_^+^-N concentrations because of NH_4_^+^-N loss via NH_3_-N volatilisation, and the rapid increase in NO_3_^−^-N content due to the high nitrification rate, especially during the maturation period. In Figure 5C, SBW composting piles exhibited the highest NH_4_^+^-N/NO_3_^−^-N ratio throughout the composting process. This may be attributed to the presence of higher degradable nitrogenous substances in the starting feedstock and higher NH_4_^+^-N concentrations recorded in such piles. Our study results revealed that the bulk agent types have a statistically significant effect (*p* = 0.0001) on the evolution of NH_4_^+^-N/NO_3_^−^-N values during FS composting. 

The NH_4_^+^-N/NO_3_^−^-N ratio has been reported by several composting studies as one of the most important compost stability or maturity indicator [39]. For instance, Bernal, et al. [72] suggested a threshold NH_4_^+^-N/NO_3_^−^-N ratio value of <0.16 for mature or stable compost. Interestingly, in this study, the recommended threshold value was reached and even exceeded by SSD piles after a very short composting period of 6 weeks (Figure 5C). However, the compost for such piles was neither mature nor stable as the composting temperatures were considerably still high, implying an active composting process. Thereafter, both temperature and NH_4_^+^-N/NO_3_^−^-N values stabilised after a composting period of 14 weeks with a NH_4_^+^-N/NO_3_^−^-N value of 0.02, which implied that the compost was then mature or stable. Surprisingly, the SCH and SBW piles attained the suggested NH_4_^+^-N/NO_3_^−^-N threshold value after a composting period of 10 and 14 weeks, which implied that the compost was mature since the composting temperatures were also relatively stable during these composting periods.

The NH_4_^+^-N/NO_3_^−^-N values recorded in this study for mature or stable compost compare well with 0.02–0.38, 0.003–5 and 0.01–20.6 published by Bernal, et al. [72], Francou, et al. [71] and Forster, et al. [73], respectively. We noted that the bulking agent types had a statistically significant effect (*p* = 0.0001) on the stability of NH_4_^+^-N/NO_3_^−^-N values and the composting period of FS. Based on NH_4_^+^-N/NO_3_^−^-N ratio maturity index, our study findings suggest that composting of FS with SBW piles reduces the composting periods of FS by 4 weeks.

### 3.8. Compost Trace Elements and Heavy Metals

The presence of high heavy metals concentrations in mature compost is one of the major concerns for recycling compost in agriculture since they have adverse effects on the plant growth and health, human health, and soil microorganisms and properties. Therefore, in our study, it was important to evaluate the concentrations of heavy metals in the initial feedstock and final compost, and these results are presented in Table 2. It can be noted in Table 2 that on a dry weight basis, all composting piles exhibited an increase in some heavy metals (Zn, Pb, Cr, Cu) and macro as well as micronutrients (Na, Mn, Mg, Ca, TP, TK) concentrations by the end of the composting process. Similar behaviour was observed by Yañez, et al. [15]. This increase in the nutritional aspects and heavy metals content is thought to have been due to the concentration effect resulting from significant degradation of matter, thus a net loss of dry weight. Surprisingly, on an ash content basis, SSD, SCH, and SBW piles exhibited a significant decrease in the concentrations of heavy metals as well as macro and micronutrients of about 12–55%, 42–74% and 51–89%, respectively, by the end of the composting process. This decrease can be attributed to the loss of heavy metals and nutrient content through leaching. During composting, a significant quantity of leachate was observed to drain from SBW piles than SSD and SCH piles. This is anticipated to have been the major reason for the significant heavy metals and nutrient content losses observed in such piles. 

Importantly, the concentrations of all toxic metals (Cd, Cr, Ni, Pb and Zn) in mature compost were far below the maximum limits (see Table 2) suggested by European Union states [74]. Further, all composting piles exhibited considerably high micro and macronutrients in the final compost, with SSD and SCH piles recording the highest (Table 2). In the present study, SSD, SCH, and SBW piles recorded NPK values of 2.7:1.1:0.7, 3.1:1.2:0.6 and 2.8:2.4:0.5, respectively, in the final compost. Such nutrient concentrations are quite sufficient for plant growth since Central Public Health and Environment Engineering Organization [75] suggested NPK content of >1% for each element in the final compost to be sufficient. 

### 3.9. Implication of Study Findings

Given the compact nature and low C/N ratio of dewatered FS, optimisation of its composting process requires an understanding and selection of the suitable bulking agent for co-composting with it. We investigated the effect of different bulking agents on the FS composting process. In this study, bulking agent types had a statistically significant effect on the evolution of composting temperatures, pH, EC, nitrogen forms, organic matter mineralisation, organic matter and nitrogen losses, maturity indices and quality of the final compost. Importantly, we observed that co-composting of FS with sawdust reduces the nitrogen losses to 2.2% and improves the plant growth index to more than 80%. N losses associated with SSD piles were considerably lower than those exhibited by coffee husks (48.2%) and brewery waste piles (72.5%). From the resource recovery perspective, our study findings suggest sawdust as a suitable bulking agent for co-composting with FS since it enhanced nutrient recovery (especially nitrogen) than other tested bulking agents. 

In our study, temperature, organic matter, C/N, NH_4_^+^-N/NO_3_^−^-N, plant growth bioassay, NH_4_^+^-N, and CO_2_-C evolution, parameters were used for assessing the FS compost maturity and stability. Based on the maturity indices suggested in the literature for these parameters, overall, the brewery waste piles reached maturity after 8 weeks, while the coffee husks and sawdust piles required more than 6 weeks of composting to reach maturity. However, this study results suggest that there is no single maturity index that can be used for assessing the composting process efficiency as well as the compost maturity since discrepancies were observed in the composting periods required to reach the maturity of the suggested indices. Therefore, it is important that more than one maturity index is used for assessing the compost maturity or stability. Based on the evolution of the physical and chemical parameters of the tested bulking agents, our study findings suggest a C/N ratio value of ≤9.6; NH_4_^+^-N/NO_3_^−^-N ratio of ≤0.02; CO_2_-C of ≤0.84 mg CO_2_-C gVS^−1^day^−1^ and PGI values of ≥81.0% as the reliable threshold values for mature and stable FS compost. 

We observed at the end of the composting period, that all piles generated pathogen-free compost (detailed results reported in a separate note elsewhere [9]) with high micro and macro-nutrient content as well as low heavy metals concentrations, which qualifies the mature FS compost attained in this study as first-class compost, according to standards suggested by European Commission [76]. These study findings are of remarkable significance as they provide insights for decentralised treatment and sustainable management of FS and other organic wastes such as sawdust, coffee husks and brewery waste in the low-and-middle income countries, especially in the rural and peri-urban communities where there is a strong practice of reusing organic waste in agriculture. Further, the results have proved that in some African communities, co-composting of FS with locally available bulking agents such as sawdust, or coffee husks may be a viable option for FS treatment at a household level or small scale. However, in such cases or scenarios, the sizes of the piles might be increased to enhance self-thermal insulation and also prolong the thermophilic phase for effective pathogen inactivation [9]. The heating up of the piles can also be enhanced by covering up the composting piles with mature compost or screen to reduce on heat losses during composting. 

## 4. Conclusions

This research study aimed at investigating the effect of locally available bulking agents (i.e., sawdust, coffee husks and brewery waste) on the faecal sludge (FS) composting process and the properties of the final FS compost. The following conclusion can be drawn based on the findings obtained:

- Irrespective of the different bulking agents used, all composting piles attained the optimum temperatures (>55 °C) and conditions suggested for effective pathogen inactivation and organic matter decomposition during composting;
- The bulking agent types have a statistically significant effect (*p* = 0.0001) on the composting periods and evolution of composting temperatures, pH, EC, organic matter mineralisation, total organic carbon, nitrogen forms, and maturity indices (i.e., Carbon dioxide (CO_2_-C), Plant Growth Index, C/N ratio, NH_4_^+^-N/NO_3_^−^-N ratio) and quality of the final compost (i.e., micro and macro-nutrients (i.e., Ca, Mg, Fe, Mn, Na) and toxic elements/heavy metals (i.e., Cu, Zn, Pb, Ni, Cr, and Cd)) during FS composting;
- From the perspective of nutrient recovery, especially nitrogen, sawdust is the most suitable bulking agent for composting with FS - as it exhibited the lowest nitrogen losses of only 2.2% compared to coffee husk (48.2%) and brewery waste (72.5%) and lowest organic matter losses, thus improving the agronomic values of the final compost as a soil conditioner;
- There is no single maturity index that can be used for assessing the composting process efficiency and compost maturity – as in our study discrepancies were observed in the composting periods required to reach the maturity of the suggested indices. Therefore, it is important that more than one maturity index is used for assessing compost maturity. Our study findings suggest a C/N ratio value of ≤9.6; NH_4_^+^-N/NO_3_^−^-N ratio of ≤0.02; CO_2_-C of ≤0.84 mg CO_2_-C gVS^−1^day^−1^ and PGI values of ≥81.0% as the reliable threshold values for mature and stable FS compost;
- Co-composting of FS with sawdust, coffee husk or brewery waste using seven days turning frequency, can generate first-class mature and stable FS compost for reuse in agriculture, within a composting period of 14 weeks. However, co-composting of FS with brewery waste can reduce the FS composting periods from 14 weeks to eight weeks;
- FS co-composting can be considered a sustainable and decentralised treatment option for FS and other organic wastes such as sawdust, coffee husks and brewery wastes in rural and peri-urban communities, especially where there is a strong practice of reusing organic waste in agriculture.

## Figures and Tables

**Table 1:** Chemical Characteristics of raw material used ^‡^.

Parameters	Dewatered FS	Sawdust	Coffee husk	Brewery Waste
pH 1:10	7.96 ± 0.15	5.66 ± 0.44	6.53 ± 0.33	6.10 ± 0.42
EC 27 °C (Sm/cm) 1:10	2.12 ± 0.42	0.40 ± 0.09	3.17 ± 0.78	1.09 ± 0.15
Moisture (%)	68.71 ± 3.80	31.20 ± 5.94	11.40 ± 2.40	67.55 ± 8.14
Bulky Density (kg/m^3^)	1066.67 ± 61.10	283.00 ± 21.21	272.00 ± 53.74	356.00 ± 33.94
Organic matter * (%)	62.17 ± 3.75	94.30 ± 2.97	87.00 ± 7.07	40.07 ± 2.12
Total organic carbon * (%)	33.83 ± 1.41	51.44 ± 2.08	52.24 ± 2.03	24.50 ± 3.54
Nitrate-N * (mg/kg)	0.05 ± 0.03	0.00 ± 0.00	0.00 ± 0.00	0.00 ± 0.00
Ammonium-N * (mg/kg)	0.53 ± 0.39	0.00 ± 0.01	0.02 ± 0.01	0.02 ± 0.00
C/N	10.11 ± 4.94	132.25 ± 69.90	17.13 ± 5.74	6.84 ± 4.81
Macro and micronutrients				
Total Kjeldah nitrogen * (%)	3.35 ± 0.29	0.39 ± 0.03	3.05 ± 0.35	3.58 ± 0.74
Total phosphorus (TP) * (g/kg)	26.9 ± 7.9	0.12 ± 0.01	0.9 ± 0.0	28.7 ± 1.4
Total potassium (TK) * (g/kg)	4.4 ± 0.3	2.0 ± 0.1	3.3 ± 0.3	3.2 ± 0.4
Calcium (Ca) * (mg/kg)	11,596.3 ± 2352.2	1445.3 ± 741.6	1433.7 ± 934.1	1667.2 ± 367.8
Magnesium Mg * (mg/kg)	4074.2 ± 729.1	575.4 ± 89.9	1292.5 ± 122.8	1654.2 ± 194.6
Iron (Fe) * (mg/kg)	6333.6 ± 888.2	983.4 ± 83.6	1746.7 ± 170.5	1021.3 ± 140.7
Manganese (Mn) * (mg/kg)	399.5 ± 249.7	7.8 ± 0.3	8.9 ± 0.6	8.0 ± 0.0
Sodium (Na) * (g/kg)	1.7 ± 0.1	0.5 ± 0.0	0.7 ± 0.1	2.2 ± 0.1
Toxic Elements/Heavy metals				
Copper (Cu) * (mg/kg)	113.7 ± 41.1	8.8 ± 1.2	11.0 ± 3.6	7.6 ± 0.6
Zinc (Zn) * (mg/kg)	448.7 ± 151.9	31.3 ± 0.6	43.4 ± 5.2	63.8 ± 3.3
Lead (Pb) * (mg/kg)	73.3 ± 29.9	8.8 ± 2.0	17.6 ± 0.9	1.2 ± 0.5
Nickel (Ni) * (mg/kg)	15.3 ± 6.8	13.9 ± 0.6	12.0 ± 0.3	14.5 ± 2.3
Chromium (Cr) * (mg/kg)	46.9 ± 36.2	13.2 ± 2.4	27.9 ± 18.2	32.6 ± 4.1
Cadmium (Cd) * (mg/kg)	0.9 ± 0.4	0.3 ± 0.3	0.7 ± 0.1	0.8 ± 0.1

^‡^ Mean ± Standard deviation (SD) of triplicates; * dry base.

**Table 2:** Nutrient and heavy metals concentrations of initial feedstock and mature compost of FS with different bulking agents.

Compost Characteristics	SSD		SCH		SBW		EU Range (2000)
	Initial	Final	Initial	Final	Initial	Final	
Bulky Density (kg/m^3^)	486.0 ± 0.0	448.0 ± 17.0	482.6 ± 2.2	485.0 ± 7.1	489.0 ± 5.7	387.5 ± 46.0	–
Organic matter (%)	75.8 ± 2.0	51.6 ± 1.2	81.4 ± 3.4	47.6 ± 0.8	85.6 ± 1.4	45.8 ± 0.5	–
Total organic carbon (%)	40.6 ± 1.2	28.6 ± 1.1	45.5 ± 2.1	25.6 ± 0.2	46.6 ± 1.0	26.9 ± 0.6	–
Total nitrogen (%)	1.4 ± 0.0	2.7 ± 0.3	2.1 ± 0.1	3.1 ± 0.3	2.7 ± 0.1	2.8 ± 0.3	–
Nitrate-N (NO_3_^−^-N) (mg/kg)	53.6 ± 22.9	787.0 ± 114.9	81.3 ± 13.5	2740.4 ± 259.0	82.0 ± 8.0	2162.7 ± 123.4	–
Ammonium-N (NH_4_^+^-N) (mg/kg)	322.1 ± 31.2	8.6 ± 0.2	205.4 ± 3.2	9.4 ± 2.2	1125.6 ± 70.4	305.3 ± 189.4	–
Macro and micronutrients							
Total phosphorus (TP) (g/kg)	6.7 ± 2.8	10.8 ± 2.4	8.8 ± 0.4	11.9 ± 2.2	21.8 ± 3.4	23.8 ± 2.2	–
Total potassium (TK) (g/kg)	2.8 ± 0.2	7.1 ± 1.2	3.2 ± 0.7	5.6 ± 1.8	3.0 ± 0.9	5.0 ± 0.9	–
Calcium (Ca) (g/kg)	6.7 ± 1.6	7.4 ± 0.4	5.2 ± 1.5	6.0 ± 1.1	5.7 ± 2.0	6.3 ± 0.4	–
Magnesium (Mg) (g/kg)	2.3 ± 0.7	2.7 ±0.5	2.8 ± 0.3	2.9 ± 0.1	2.0 ± 0.4	2.2 ± 0.3	–
Iron (Fe) (mg/kg)	2175.1 ± 421.7	2955.7 ± 48.2	2974.8 ± 774.6	3146.2 ± 374.9	2641.1 ± 900.8	2094.6 ± 395.9	–
Manganese (Mn) (mg/kg)	109.5 ± 9.7	204.3 ± 12.0	180.0 ± 52.8	364.0 ± 11.4	114.9 ± 46.2	201.3 ± 30.4	–
Sodium (Na) (g/kg)	1.0 ± 0.0	1.4 ± 0.1	0.9 ± 0.2	1.1 ± 0.3	0.8 ± 0.2	1.2 ± 0.1	–
Toxic Elements/Heavy metals							
Copper (Cu) (mg/kg)	30.8 ± 6.6	41.9 ± 15.0	47.4 ± 2.8	53.9 ± 15.7	35.6 ± 4.3	48.4 ± 7.4	70–600
Zinc (Zn) (mg/kg)	245.3 ± 91.0	349.4 ± 12.7	200.7 ± 72.3	337.6 ± 30.5	231.6 ± 78.5	313.5 ± 4 3.8	210–4000
Lead (Pb) (mg/kg)	39.5 ± 4.7	38.3 ± 7.7	33.3 ± 11.1	44.6 ± 4.2	46.4 ± 6.9	41.1 ± 7.4	70–1000
Nickel (Ni) (mg/kg)	8.1 ± 0.8	12.8 ± 0.0	10.8 ± 4.3	9.1 ± 2.6	13.7 ± 3.5	12.0 ± 3.0	20–200
Chromium (Cr) (mg/kg)	39.5 ± 1.7	54.2 ± 0.7	56.2 ± 35.7	58.5 ± 15.9	52.6 ± 22.7	63.2 ± 11.2	70–200
Cadmium (Cd) (mg/kg)	0.51 ± 0.35	0.5 ± 0.1	0.57 ± 0.09	0.4 ± 0.1	0.8 ± 0.4	0.6 ± 0.1	0.7–10

Compost of faecal sludge with Brewery waste-SBW, Coffee husks-SCH, and Sawdust-SSD.

## Data Availability

Data supporting this study are included within the article and supporting materials.

## Supplementary Materials

The following are available online at https://www.mdpi.com/article/10.3390/ijerph191710592/s1, Table S1: Summary of composting temperatures recorded from piles section during the composting of FS with different bulking agents. Figure S1: Changes in (A) Ash content, (B) Total organic carbon losses, (C) Nitrogen Losses and (D) Organic matter losses during the composting of Faecal Sludge-FS with Brewery waste-SBW, Coffee husks-SCH, and Sawdust-SSD. Table S2: Analytical methods and laboratory instruments used for analyzing compost samples for physical and chemical properties. Supporting Data. References [27,29,30,31,33,34,35,36,58] are cited in the supplementary materials.
