# Supplementary material for: Recycling of Faecal Sludge: Nitrogen, Carbon and Organic Matter Transformation during Co-Composting of Faecal Sludge with Different Bulking Agents
Source: Int J Environ Res Public Health. 2022 Aug 25;19(17):10592. doi: 10.3390/ijerph191710592 (PMC9518209; doi:10.3390/ijerph191710592)
Supplement: Supplementary file 1 [file ijerph-19-10592-s001.zip › ijerph-1825979-supplementary.pdf]

Supplementary Information

Recycling of Faecal Sludge: Nitrogen, Carbon and Organic Matter Transformation during Co-Composting of Faecal Sludge with Different Bulking Agents

Musa Manga [1,2,3,*], Barbara E. Evans [2], Tula M. Ngasala [4] and Miller A. Camargo-Valero [2,5]

1 The Water Institute at UNC, Department of Environmental Sciences and Engineering, Gillings School of Global Public Health, University of North Carolina at Chapel Hill, 357 Rosenau Hall, 135 Dauer Drive, Chapel Hill, NC 27599, USA
2 School of Civil Engineering, University of Leeds, Leeds LS2 9JT, UK
3 Department of Construction Economics and Management, College of Engineering, Design, Art and Technology (CEDAT), Makerere University, Kampala P.O Box 7062, Uganda
4 Department of Civil and Environmental Engineering, Michigan State University, East Lansing, MI 48823, USA
5 Departamento de Ingeniería Química, Universidad Nacional de Colombia, Campus La Nubia, Manizales 170003, Colombia
* Correspondence: mmanga@email.unc.edu or musa.manga@mak.ac.ug

Table S1. Summary of composting temperatures recorded from piles section during the composting of FS with different bulking agents.

Pile	Pile Section	Composting Period to reach T ≥ 55°C (Days)	Composting Period with T ≥ 55°C (Days)	Composting Period to attain T-max (days)	T-max (°C)	Composting Period to drop T ≤ 45°C (Days)	Total Composting period
SSD							
	Top	16	46	20	58.2	71	
	Middle	16	40	17	56	72	
	Bottom	NT	NT	19	52.8	54	
	Sides	17	23	20	56.9	70	
	mean	NT [i]	NT [ii]	51 [iii]	53.5 [iv]	69 [v]	109
SCH							
	Top	16	56	52	72	107	
	Middle	5	47	52	67	104	
	Bottom	NT	NT	63	53	64	
	Sides	16	56	52	71	100	
	mean	41 [i]	54 [ii]	52 [iii]	65 [iv]	99 [v]	136
SBW							
	Top	14	27	35	69	47	
	Middle	11	25	27	70.2	47	
	Bottom	19	12	24	68.3	41	
	Sides	15	26	27	68.7	43	
	mean	16 [i]	22 [ii]	19 [iii]	67 [iv]	44 [v]	57

NT:-Not attained. [i] Represents the composting period at which the mean composting temperature of all the monitored pile sections (top, middle, bottom and sides) reached temperature ≥ 55°C. [ii] Represents the composting period with which the mean composting temperature of all the monitored pile sections (top, middle, bottom and sides) was ≥ 55°C. [iii] Represents the composting period at which the mean composting temperature of all the monitored pile sections (top, middle, bottom and sides) reached the maximum temperature. [iv] Represents the maximum temperature recorded by the mean composting temperature of all the monitored pile sections (top, middle, bottom and sides) at a given composting period. [v] Represents the composting period at which the mean composting temperation of all the monitored pile sections (top, middle, bottom and sides) dropped to temperatues ≤ 45°C.

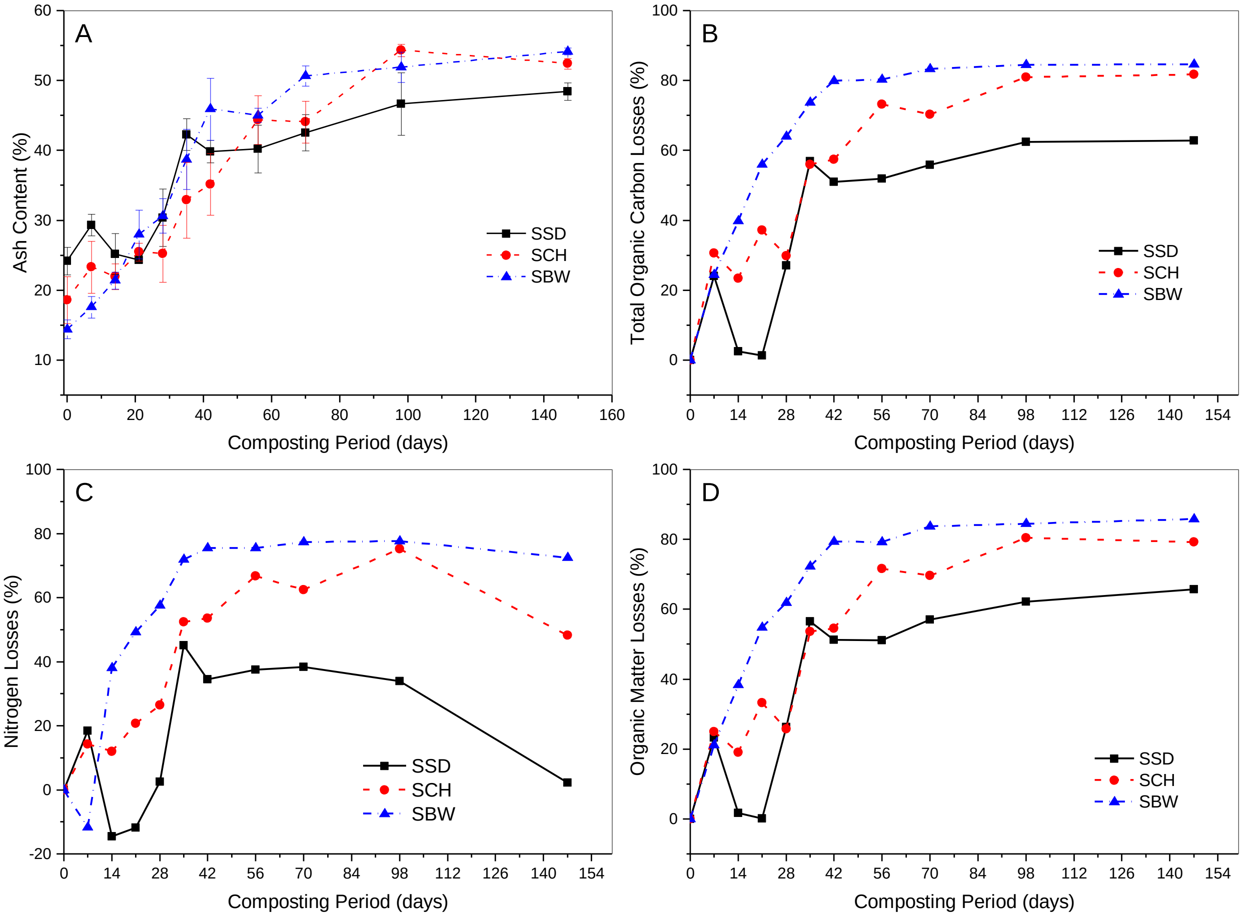

Figure S1. Changes in (A) Ash content, (B) Total organic carbon losses, (C) Nitrogen Losses and (D) Organic matter losses during the composting of Faecal Sludge – FS with Brewery waste – SBW, Coffee husks – SCH, and Sawdust – SSD. Error bars represent the standard error of *n* = 2.

Table S2: Analytical methods and laboratory instruments used for analyzing compost samples for physical and chemical properties.

Analysis	Method used	Instruments used (Model, Producer, Origin)	Reference
Composting Temperature		Stainless steel compost thermometer. TFA, D-Wertheim, Model 19.2008, UK	
Moisture Content	Gravimetric method	Gallenkamp Hot Box Bench Top Laboratory Oven with fan, Model CHF097.XX2.5; London, UK	[27]
Organic matter/ Volatile solids	Gravimetric method	Heavy Duty Muffle Furnace, 240V FA1730-1, Thermolyne thermos scientific, USA	[27]
pH	Potentiometric method	pH electrode of HACH sensION+ MM374 Multi-Parameter Benchtop meter, USA	58
EC	Potentiometric method	EC probe of HACH sensION+ MM374 Multi-Parameter Benchtop meter, USA	[58]
Total organic carbon (%)	Oxidation using Potassium dichromate		[27,29]
Nitrogen (%)	Semi-micro Kjeldahl method	Auto distillation unit, FOSS Kjeltec™ 8200; Hoganas, Sweden	[27,30]
Nitrate-N (NO_3^--N) (mg/kg)	Spectrophotometrically by Sodium salycilatum acid colorimetric method	DR6000 Benchtop Spectrophotometer, HACH, USA<br>Absorbance measured at 419 nm wavelength.	[27,31]
Ammonium-N (NH_4^+-N) (mg/kg)	Spectrophotometric methods	DR6000 Benchtop Spectrophotometer, HACH, USA<br><br>Absorbance measured at 655nm wavelength	[27,31]
CO_2-C evolution	Öhlinger [33] soil respiration techniques		[34,35]

**Macro and micronutrients**			
Total phosphorus (TP) (g/kg)	Wet -digestion method of sample preparation, Ascorbic Acid method, Spectrophotometric methods	DR6000 Benchtop Spectrophotometer, HACH, USA<br><br>Absorbance measured at 880nm wavelength	[27]
Total potassium (TK) (g/kg)	Wet digestion method of sample preparation, Flame photometry	Flame photometer: - Model 420 Flame photometer, Sherwood scientific, UK<br><br>Measured at **766** nm a wavelength	[27]

Analysis	Method used	Instruments used (Model, Producer, Origin)	Reference
Calcium (Ca) (g/kg)	Wet -digestion method of sample preparation, Atomic Absorption Spectrometry	Atomic Absorption Spectrophotometer – Agilent 240Z AA (200 Series AA) with Programmable Sample Dispenser (PSD) 120 and Graphite Tube Atomizer (GTA) 120, Model, AA-01-0400, Agilent Technologies, Canada<br><br>Measured at **422.7** nm wavelength	[27,36]
Magnesium (Mg) (g/kg)	Wet -digestion method of sample preparation, Atomic Absorption Spectrometry	Atomic Absorption Spectrophotometer – Agilent 240Z AA (200 Series AA) with Programmable Sample Dispenser (PSD) 120 and Graphite Tube Atomizer (GTA) 120, Model, AA-01-0400, Agilent Technologies, Canada<br><br>Measured at **285.2** nm wavelength	[36]
Iron (Fe) (mg/kg)	Wet -digestion method of sample preparation, Atomic Absorption Spectrometry	Atomic Absorption Spectrophotometer – Agilent 240Z AA (200 Series AA) with Programmable Sample Dispenser (PSD) 120 and Graphite Tube Atomizer (GTA) 120, Model, AA-01-0400, Agilent Technologies, Canada<br><br>Measured at **248.3** nm wavelength	[36]
Manganese (Mn) (mg/kg)	Wet -digestion method of sample preparation, Atomic Absorption Spectrometry	Atomic Absorption Spectrophotometer – Agilent 240Z AA (200 Series AA) with Programmable Sample Dispenser (PSD) 120 and Graphite Tube Atomizer (GTA) 120, Model, AA-01-0400, Agilent Technologies, Canada<br><br>Measured at **279.5** nm wavelength	[36]
Sodium (Na) (g/kg)	Wet digestion method of sample preparation, Flame photometry	Flame photometer: - Model 420 Flame photometer, Sherwood scientific, UK<br><br>Measured at **589** nm wavelength	[27]
**Toxic Elements/ Heavy metal**			
Copper (Cu) (mg/kg)	Wet -digestion method of sample preparation, Atomic Absorption Spectrometry	Atomic Absorption Spectrophotometer – Agilent 240Z AA (200 Series AA) with Programmable Sample Dispenser (PSD) 120 and Graphite Tube Atomizer (GTA) 120, Model, AA-01-0400, Agilent Technologies, Canada<br><br>Measured at **324.8** nm wavelength	[36]

Analysis	Method used	Instruments used (Model, Producer, Origin)	Reference
Zinc (Zn) (mg/kg)	Wet -digestion method of sample preparation, Atomic Absorption Spectrometry	Atomic Absorption Spectrophotometer – Agilent 240Z AA (200 Series AA) with Programmable Sample Dispenser (PSD) 120 and Graphite Tube Atomizer (GTA) 120, Model, AA-01-0400, Agilent Technologies, Canada<br><br>Measured at **213.9** nm wavelength	[36]
Lead (Pb) (mg/kg)	Wet -digestion method of sample preparation, Atomic Absorption Spectrometry	Atomic Absorption Spectrophotometer – Agilent 240Z AA (200 Series AA) with Programmable Sample Dispenser (PSD) 120 and Graphite Tube Atomizer (GTA) 120, Model, AA-01-0400, Agilent Technologies, Canada<br><br>Measured at **283.3** nm wavelength	[36]
Nickel (Ni) (mg/kg)	Wet -digestion method of sample preparation, Atomic Absorption Spectrometry	Atomic Absorption Spectrophotometer – Agilent 240Z AA (200 Series AA) with Programmable Sample Dispenser (PSD) 120 and Graphite Tube Atomizer (GTA) 120, Model, AA-01-0400, Agilent Technologies, Canada<br><br>Measured at **232.0** nm wavelength	[36]
Chromium (Cr) (mg/kg)	Wet -digestion method of sample preparation, Atomic Absorption Spectrometry	Atomic Absorption Spectrophotometer – Agilent 240Z AA (200 Series AA) with Programmable Sample Dispenser (PSD) 120 and Graphite Tube Atomizer (GTA) 120, Model, AA-01-0400, Agilent Technologies, Canada<br><br>Measured at **357.9** nm wavelength	[36]
Cadmium (Cd) (mg/kg)	Wet -digestion method of sample preparation, Atomic Absorption Spectrometry	Atomic Absorption Spectrophotometer – Agilent 240Z AA (200 Series AA) with Programmable Sample Dispenser (PSD) 120 and Graphite Tube Atomizer (GTA) 120, Model, AA-01-0400, Agilent Technologies, Canada<br><br>Measured at **228.8** nm wavelength	[36]
